# HISTORY: MINNESOTA FAMILY WORKSHOP TO SAVVY CAREGIVER: CENTRALITY OF SELF-EFFICACY

**DOI:** 10.1093/geroni/igac059.1700

**Published:** 2022-12-20

**Authors:** Kenneth Hepburn

**Affiliations:** Emory University, Atlanta, Georgia, United States

## Abstract

This presentation will trace the development of the Savvy Caregiver program from its beginnings as the Center for Nursing Research-supported Minnesota Family Workshop (1993; PI: Sharon Ostwald) through a further test as the Partners in Caregiving Program (1997; PIs: Hepburn 1RO1NR04517-01) to the Alzheimer’s Association-supported Savvy program (1997; PIs Hepburn & Lewis). Three main developments occurred over this period: the program solidified its identity as a caregiver training program and its mechanism of action as that of self-efficacy development through an active learning approach; it sharpened its focus by concentrating on the principal family caregiver (moving away from a broader family approach and eliminating concurrent care recipient programming); and it developed interventionist training materials and programs to enable broader reach. Since 2002, Savvy has enjoyed wide dissemination as an evidence-based in-person psychoeducation program, fostered by support to sponsoring organizations by the Administration on Community Living.

